# Malignant Disease of the Uterus and Vagina

**Published:** 1874-01

**Authors:** H. D. Vosburgh

**Affiliations:** Lyons, N. Y.


ART. II.—*Malignant Disease of the Uterus and Vagina.* By H. D. VOSBURGH, M. D., Lyons, N. Y.

Mrs. M——, of Phelps, Ontario Co., consulted me Jan. 4, 1872. She is a tall anæmic woman, though well nourished, 53 years of age, giving the following history. She was married at the age of

37, and has given birth to three children, the youngest born when she was 45. Her menstrual periods were always regular till after the birth of the last child, when the intervals became longer and soon the menstrual flow ceased entirely. Four years after she was called to nurse a sick friend, and for a few weeks was overburdened, broken of rest at night, ascending and descending long flights of stairs, several times a day, and doing such other duties as are required in tenderly nursing sick patients until she was thoroughly exhausted. At this time there appeared a bloody discharge per vaginum. Subsequent rest did not restore her strength nor arrest the hemorrhage. The latter, though at first slight, after continuing for several weeks, became a little more profuse and quite alarmed her. In her anxiety she was urged by friends, and did consult a Gynæcological quack, who persuaded her to submit to frequent applications of *caustic potash* through a vaginal speculum, though in his maudlin condition he doubtless was very indefinite in his point of application. These were attended by burning vaginal pains, and followed by feverishness and soreness to such an extent as to compel rest for a few days immediately after. After several "Treatments" the hemorrhage became intermitting—the interval from two days to a week in duration, when it would recur again in gushes, attended by pains like labor pains.

When I first saw her the caustics had been discontinued two months. On making an examination I found the vagina to measure just two and one-half inches in length, and to terminate abruptly as though tied up with a string. Through this stricture I carefully traced a small passage with a fine silver probe, but could not discover the uterus except by rectal examination, when I could feel it high up in the pelvis. In this way too I could feel the point of my probe nearly reaching the womb and only separated from my finger by the normal amount of tissue. I advised tonics and rest and a discontinuing of any further local interference, hoping the hemorrhage would cease spontaneously. About two months after this she consulted me again. The hemorrhage had ceased, but there was a feeling of weight and pressure in the pelvis, with difficult urination. A vaginal examination revealed a tumor of a doughy feeling, nearly filling the pelvis and pressing backward upon the rectum and forward upon the urethra. I diagnosed it to be the vagina above the stricture distended by fluid, but feeling uncertain what course to pursue advised consultation. The conclusion arrived at by an eminent medical gentleman, after careful and thorough examination, was complete retroversion of the uterus backwards and filled with menstrual fluid. From this view I strongly dissented, but as no plan of treatment was advised, except to watch the case, I was content to do so.

The tumor continued to enlarge until it rested upon the floor of the pelvis and could be felt above the pubis, crowding the bladder so far upward that the catheter, which had to be relied upon entirely, passed upwards and forwards eight inches before reaching it. Her general health began rapidly to decline. The urine was loaded with mucous and pus, and her sufferings were constant and severe.

Sept. 2.—Professor J. F. Miner, of Buffalo, saw her in consultation with me. As indistinct fluctuation could be felt in the tumor it was determined to make exploration with the trochar. Upon introducing it two quarts of dark bloody fluid escaped and the tumor was collapsed. This fluid had no fœtor whatever, nor was there any thing in it or the patient which lead us to suspect malignant trouble.

The unfavorable symptoms existing prior to this time now rapidly subsided, and the patient seemed in a fair way to recover. After a fortnight slight hemorrhage began and it was soon apparent that the walls of the vagina which had constituted this tumor were breaking down. The finger now could be readily carried through the opening made by the trochar—the os uteri nearly complete could be felt in its normal position.

The hemorrhage soon became alarming and was only arrested by the most potent styptics. The vaginal discharges were very offensive and the distruction of tissues rapid.

She died exhausted Oct. 18.

Post-mortem 32 hours after death. Rigor unortis well marked; abdomen distended and tympanitic; thighs emphysematous; on opening abdomen found the peritonium every where rough and thickened and filled with horribly offensive pus and broken down tissue; bladder small, thickened, and firmly adherent to abdominal walls; lower and left border of omentum thickened and easily crushed down under the finger. The upper portion of the vagina which had formed the walls of the sack, heretofore described, was entirely destroyed, leaving a free communication into the abdominal cavity. The uterus was found perfectly in place, considerably enlarged, and the lower one-half of the neck entirely destroyed, A section showed its internal surface thrown into folds and knobs and its villi so much elongated as to have the appearance of a surface covered with moss.

This case had baffled the diagnostic skill of several eminent medical practitioners, nor did any one even suspect it to be malignant till near its sad termination.

The question arises whether it had this malignant character *de novo*, or whether it was not called into existence by the regular and indiscriminate use of the caustics in the hands of this reckless quack.

